# Combining infliximab, anti-MAP and hyperbaric oxygen therapy for resistant fistulizing Crohn's disease

**DOI:** 10.4155/fso.15.77

**Published:** 2015-09-28

**Authors:** Gaurav Agrawal, Thomas Borody, Robert Turner, Sharyn Leis, Jordana Campbell

**Affiliations:** 1 Centre for Digestive Diseases, Level 1/229 Great North Rd, Five Dock NSW 2046, Australia; 2 Prince of Wales Hospital, 1st Floor, Edmund Blacket Building, Randwick NSW 2031, Australia

**Keywords:** Crohn's disease, fistula, hyperbaric oxygen therapy, infliximab, *Mycobacterium avium ss paratuberculosis*

## Abstract

**Background:**

Fistulizing Crohn's disease (CD) presents a therapeutic challenge as fistulae are notoriously difficult to heal. *Mycobacterium avium ss paratuberculosis* (MAP) treatment in CD is gaining attention.

**Aim:**

We evaluated healing of CD fistula(e) using a novel combination therapy.

**Study:**

Nine consecutive patients who failed to heal fistulae on conventional treatment including anti-TNF, were treated with at least three doses of infliximab, 18–30 courses of hyperbaric oxygen therapy and anti-MAP antibiotics comprising rifabutin, clarithromycin and clofazimine.

**Results:**

All patients achieved complete healing of fistulae by 6–28 weeks and follow-up for mean 18 months.

**Conclusion:**

Combining infliximab, hyperbaric oxygen therapy and anti-MAP, seems to enable healing of recalcitrant fistulae and although a small case series, all nine patients achieved complete healing.

## Abstract (lay abstract)

**Lay abstract:** Nine consecutive patients with Crohn's disease and fistulae were treated with a special combination of infliximab, numerous hyperbaric oxygen sessions and combined antibiotics including rifabutin, clofazimine and clarithromycin as the base antibiotic combination – called anti-*Mycobacterium avium ss paratuberculosis* (MAP) therapy. At between 6 weeks and 6 months all fistulae healed – included rectovaginal – so that the skin was dry and there was no discharge and no need to wear a pad. Their Crohn's symptoms of diarrhea, urgency and bleeding also resolved. Continuation with anti-MAP therapy alone maintained healing although one patient who ceased the anti-MAP therapy had a relapse.

**Figure 1.**
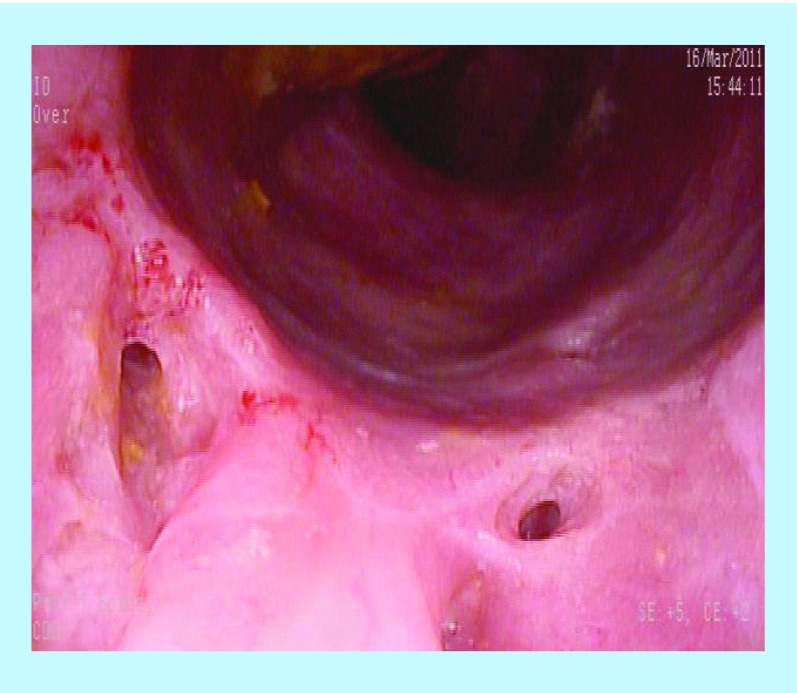
Pretreatment – multiple fistulae and inflammatory changes.

**Figure 2.**
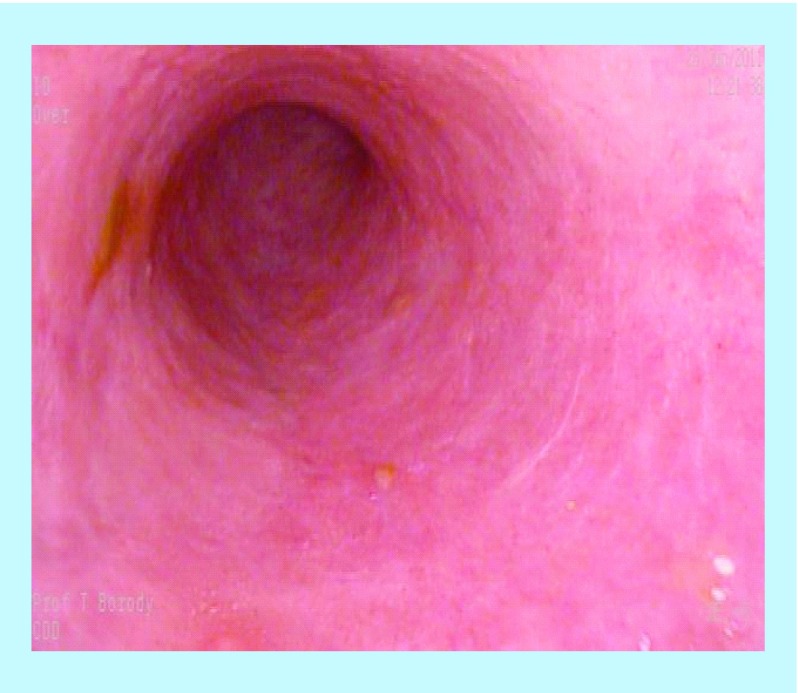
Post-treatment – endoscopic healing of previous fistulae.

**Figure 3.**
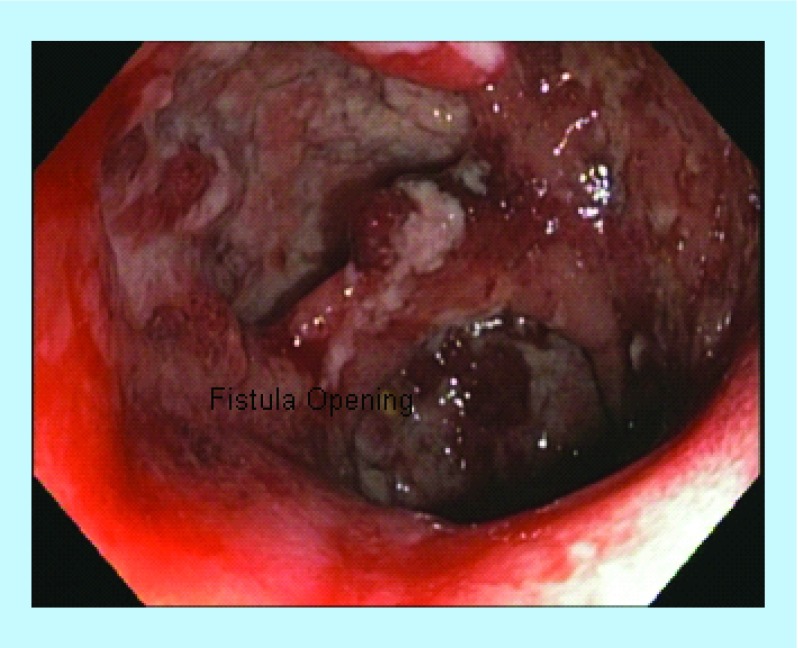
Pretreatment – rectovaginal fistula.

**Figure 4.**
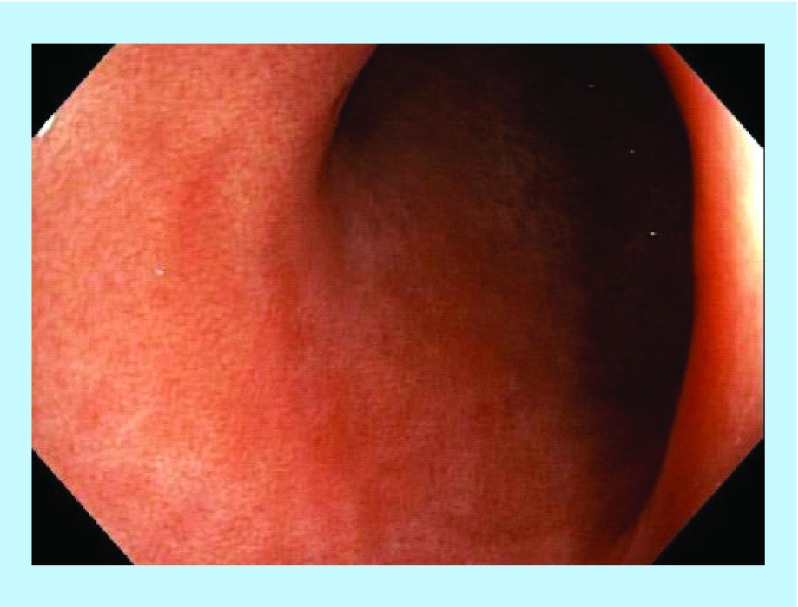
Post-treatment – endoscopically healed.

**Figure 5.**
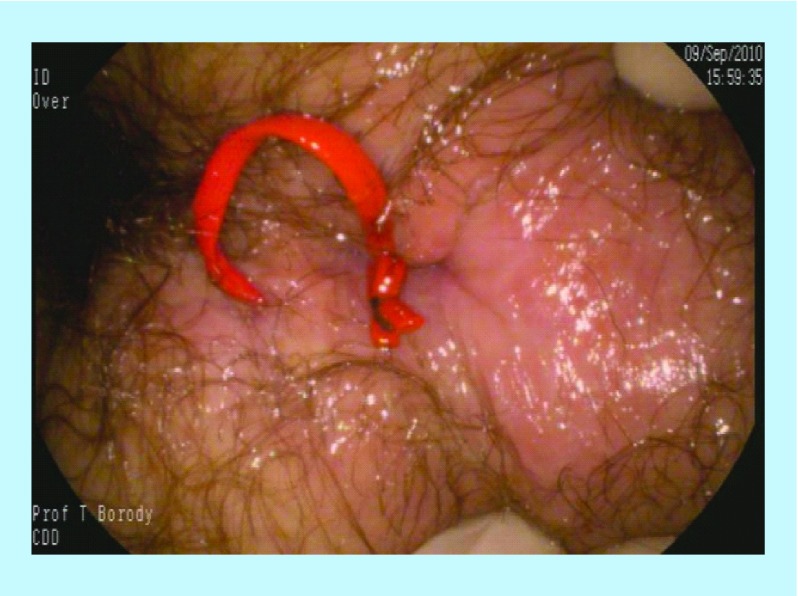
Pretreatment – perianal fistula.

**Figure 6.**
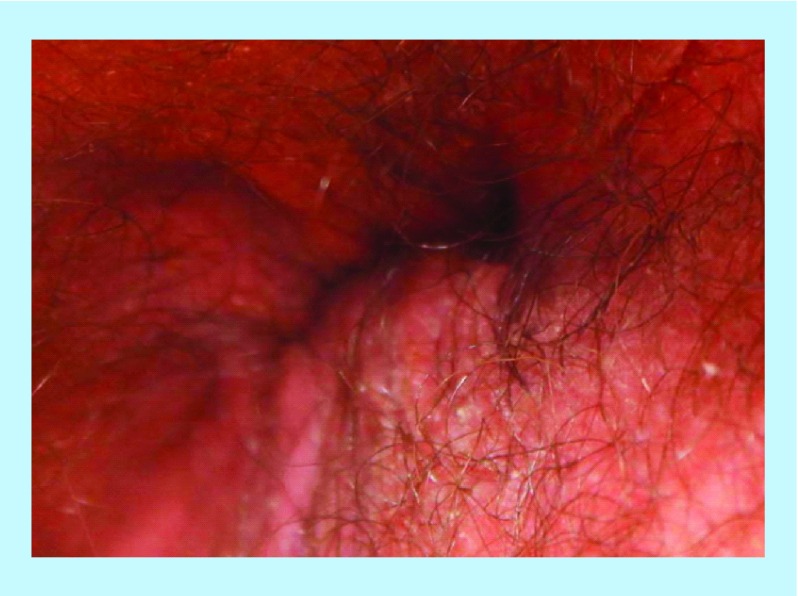
Post-treatment – healed fistula with remnant fissure.

**Figure 7.**
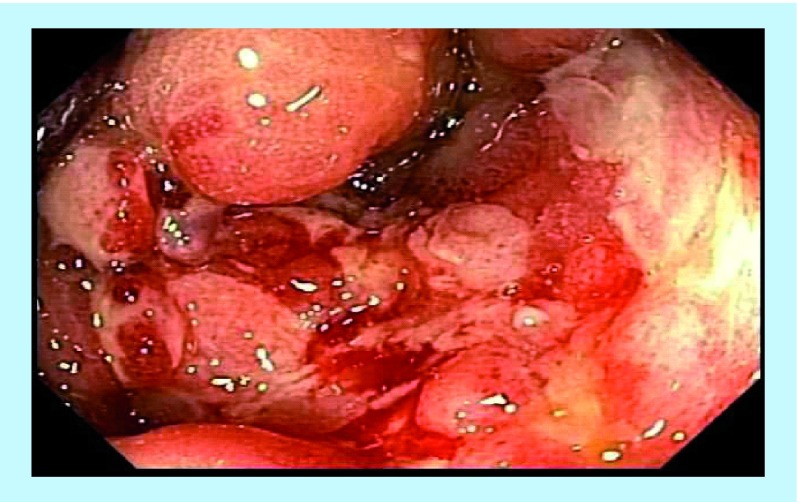
Pretreatment – rectal inflammation.

**Figure 8.**
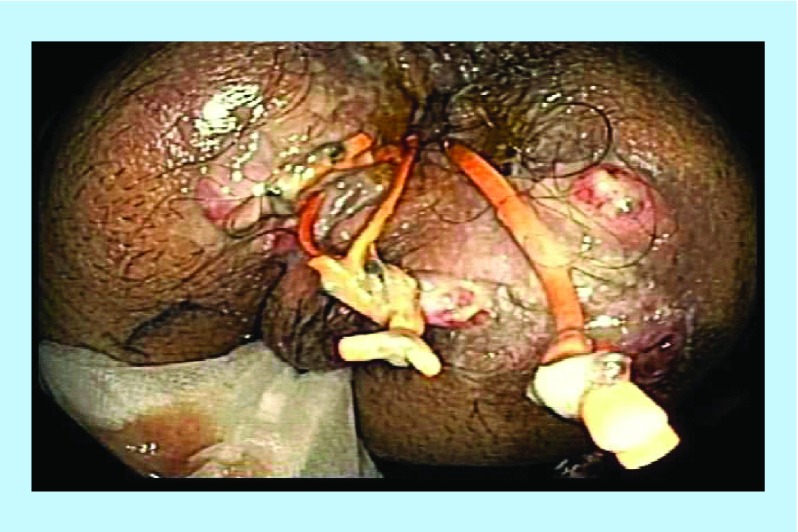
Pretreatment – multiple fistulae.

**Figure 9.**
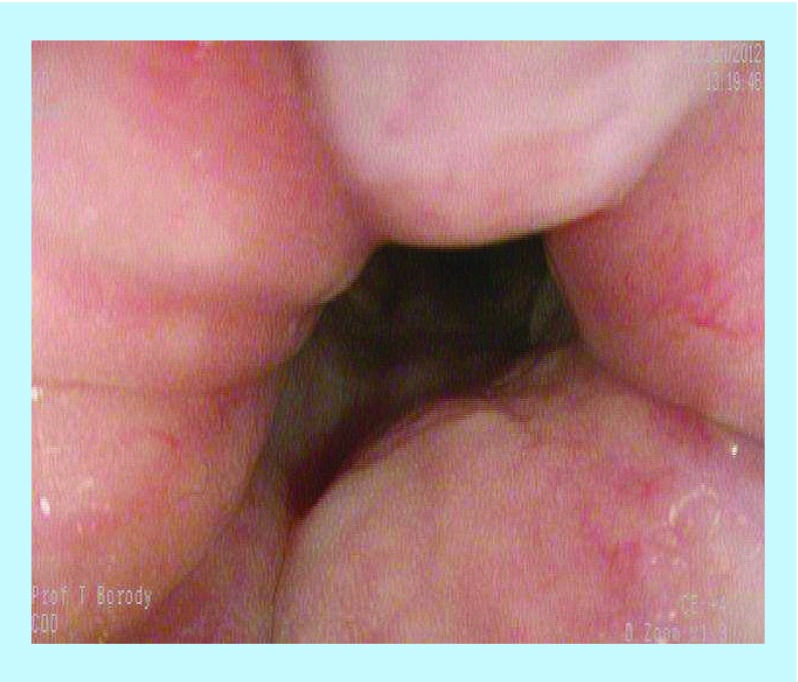
Post-treatment – healed mucosa.

**Figure 10.**
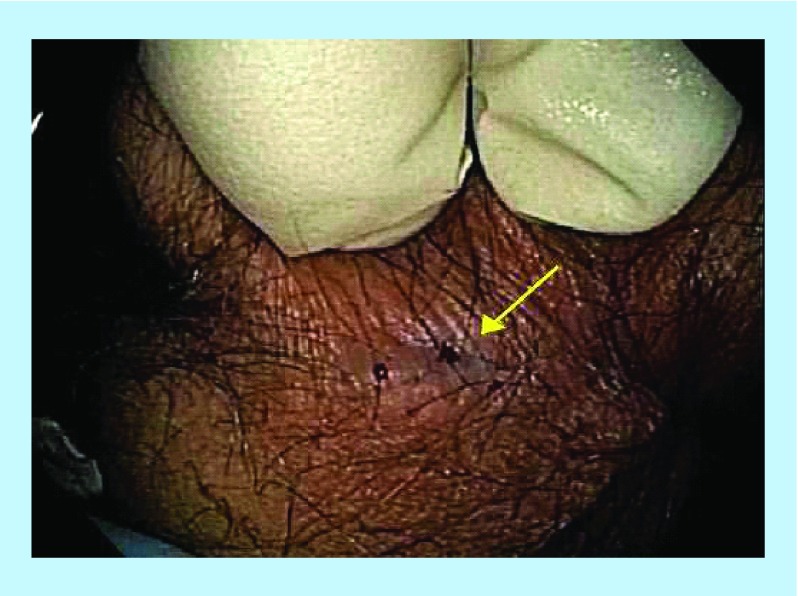
Post-treatment – healed fistula opening.

**Figure 11.**
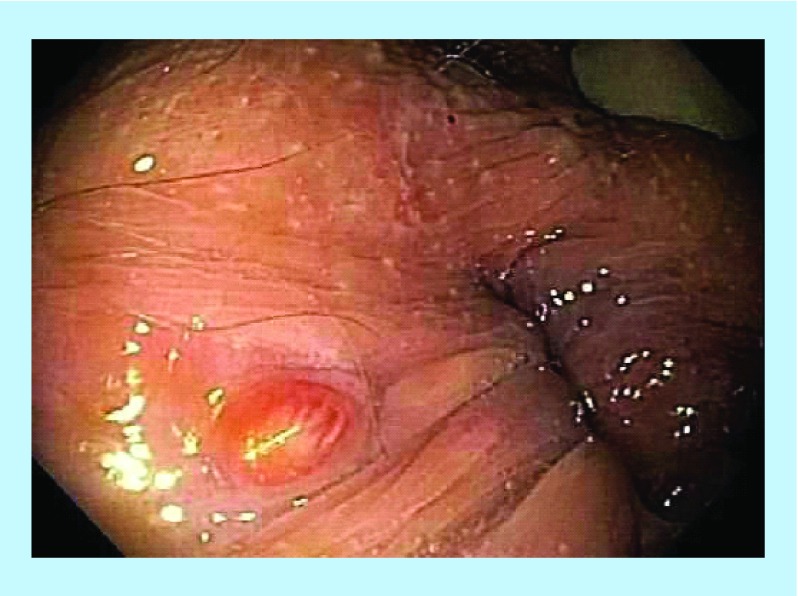
Pretreatment – fistula opening.

**Figure 12.**
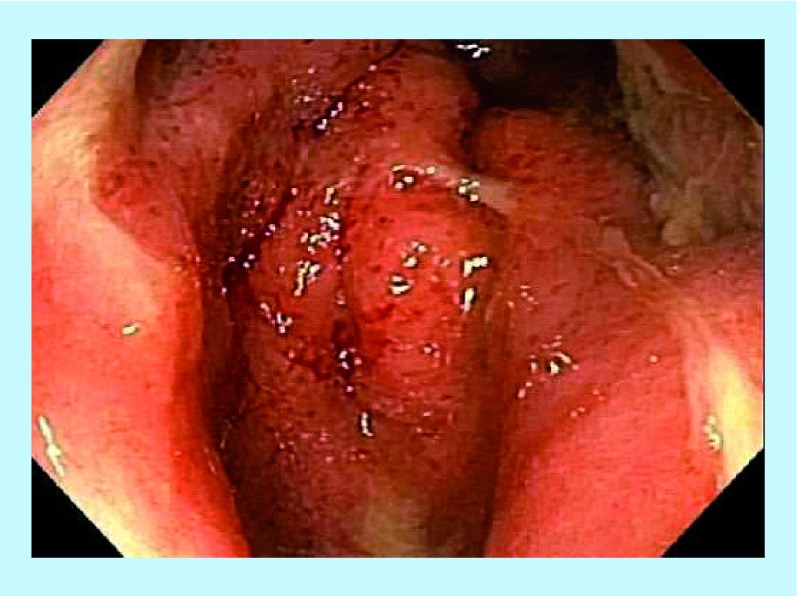
Pretreatment – rectal inflammation opening.

**Figure 13.**
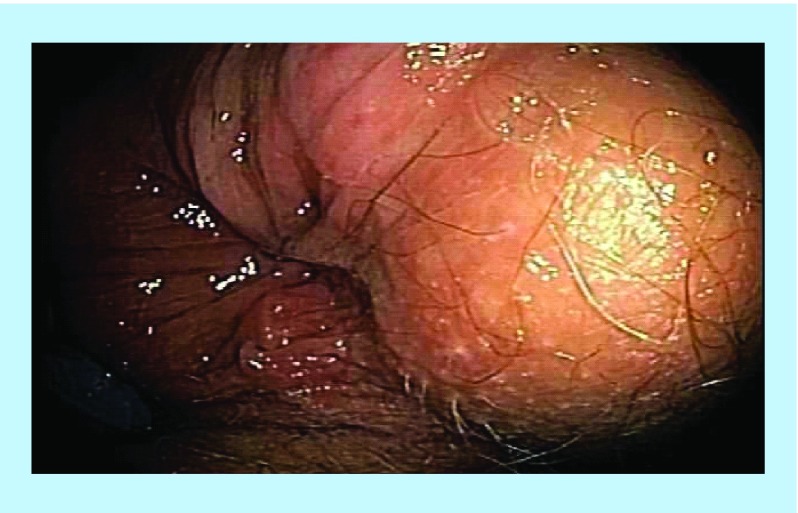
Post-treatment – healed opening.

**Figure 14.**
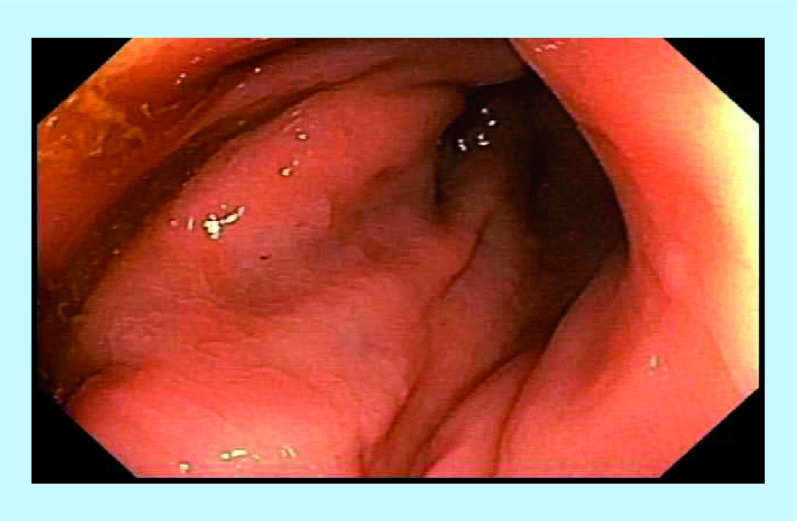
Post-treatment – healed rectal mucosa.

Due to the transmural nature of Crohn's disease (CD), approximately 20–40% of patients develop perianal fistulae [1,2], which is a distressing and potentially serious complication of the disease. Involvement may be severe and debilitating characterized by deep fistulae threatening the sphincter, numerous tracts, adjacent organ involvement and severe rectal disease [2], which restrict daily functioning, sexual activity [3] and overall quality of life [4,5]. Clinical features include painful induration, discharge, incontinence, general malaise and fever. Spontaneous healing of fistulae is rare, and they are frequently recalcitrant to treatment. Immunomodulatory agents (e.g., infliximab) represent a mainstay of fistulae treatment and achieve initial healing rates of around 60% [6,7]. However their long-term effectiveness has been questioned in prospective, placebo-controlled trials [7,8].

A possible causative link between Crohn's disease and *Mycobacterium avium ss paratuberculosis* (MAP) has been debated vigorously and for a long time. It originated from early observed similarities between CD and Johne's Disease, the enteric MAP infection in cattle. In select institutions worldwide, antibiotic combination (anti-MAP) therapy has been offered as a treatment option for CD, often with striking results [9]. Preliminary findings have further demonstrated efficacy in achieving fistula healing in patients with severe CD, with cure rates of 66.6% (4/6) in a small case series, and partial healing in a further patient (1/6) [10]. Although a randomized trial by Selby *et al*. of anti-MAP [11] showed there was no efficacy in the long-term use of anti-Map in Crohn's, at 16 weeks it was reported by Selby as being highly effective (p = 0.02). However, his trial received published criticism in the Lancet as original calculations were flawed [12]. Indications for anti-MAP treatment generally include failure or poor response to other CD therapies and particularly in those facing colectomy. Contraindications include allergy to its various components and leucopenia.

Hyperbaric oxygen therapy (HBOT), the inhalation of 100% oxygen at a pressure of >1 ATM, is an established treatment for accelerating healing of chronic nonhealing wounds, for example, diabetic foot ulcers [10,13]. It presumably works by increasing tissue oxygen levels, relieving hypoxia and altering inflammatory pathways [14]. Given such possible mechanisms of action, its value as a potential treatment option for complex perianal CD has been identified [1]. HBOT has independently achieved complete healing rates of 45% and partial healing rates of 42.5% in a systematic meta-analysis [14], presumably through the targeting of anaerobic bacteria colonizing fistulae [7]. Patients are assessed by a specialist trained in HBOT to identify such comorbidities as inner ear pathology and asthma, among others.

Given the partial success individually of infliximab, HBOT and anti-MAP the potential of combining all three was explored in CD patients with treatment-refractory fistulae. Some of these patients had reached the stage of being offered colectomy.

## Methods

Nine consecutive patients with severely active CD and intractable fistulae were prospectively followed. All were nonresponsive to standard CD treatments, including corticosteroids, 5 amino salicylates (5ASA), thiopurines, methotrexate and biologics. All had documented CD and fistulae and after obtaining signed consent all were treated with a combination of:

Infliximab 5 mg/kg (three to eight infusions; mean: 4.4 infusions);
Continuous anti-MAP therapy (see Table 1 for details);
HBOT concurrently 18–30 daily sessions at 2.0–2.4 ATM (2–2.4 bar) for 90 min (mean: 21.8 sessions) in a monoplace or multiplace chamber.

Response was assessed by a change in clinical symptoms – particularly complete fistula healing with dry, nonoozing skin, colonoscopic findings of healed mucosa and pre- and post-treatment MRI improvement. Given an accuracy of 90%, MRI is considered the preferred imaging modality for detecting and classifying fistulizing perianal disease, and was used in each case to confirm fistulae healing [15].

## Results

### Case 1

A 24-year-old male with long-standing CD presented with multiple rectum-to-rectum fistulae and inflammatory changes (Figure 1).

MRI confirmed the presence of multiple (>3) perirectal abscesses, the largest of these being 1 cm in diameter. The patient had failed previous numerous treatments. He underwent three infusions of infliximab and 30 sessions of HBOT, while being treated with combination anti-MAP (Table 1). At 3 months post-combination therapy, the patient was in asymptomatic clinical remission, with fistulae and abscesses undetectable on MRI (Figure 2). Healing persisted at 10 month colonoscopy and remains well at 2 years follow-up on maintenance anti-MAP therapy alone.

### Case 2

A 32-year-old male presented with a 3-year history of CD and a high left-sided posterior, and right anterior fistulae, which required ‘setons’. Symptoms included diarrhea, urgency and perianal discharge and tenderness. On colonoscopy, inflammation extended throughout the entire colon, with Crohn's ulcerations destroying areas of haustrations. The patient underwent three infliximab infusions, 21 HBOT sessions and anti-MAP therapy (Table 1). At 6 weeks review both fistulae healed and the skin was dry and the MRI was normal showing scarring only. Setons were removed during the latter stages of the HBOT therapy. Initially elevated inflammatory markers fell to within normal ranges. The patient continues well on anti-MAP therapy.

### Case 3

A 32-year-old female presented with a 22-year history of CD, complicated by a rectovaginal fistula (Figure 3). The patient's symptoms included severe dyspareunia and vaginal passage of gas especially during bowel evacuation. She also reported frequent loose motions, urgency, tenesmus, rectal bleeding and mucus. The patient had previously failed various treatments including a fistula treatment with infliximab alone. On colonoscopy, active, ulcerating CD was confirmed, with a visible fistula opening (Figure 3). Combination therapy consisted of four infliximab infusions, 21 HBOT sessions and anti-MAP therapy (Table 1). After 3 months, the patient reported increased energy, resolution of bowel symptoms and the return of previously absent menses. Complete rectal and vaginal fistula healing was observed on colonoscopy (Figure 4). Subsequent MRI showed disappearance of the original abnormalities in the anorectal area with no gas-containing locus and absence of the rectovaginal fistula. However, feeling so well at follow-up she decided to discontinue anti-MAP therapy and her symptoms recurred and fistula reopened.

### Case 4

A 31-year-old male with a 10-year history of CD presented with lower abdominal and rectal pain, bloating, flatulence, fecal urgency and soiling. On colonoscopy, the patient had a posterior anal fissure and an intersphincteric fistula (Figure 5). He had previously failed infliximab and later surgery and was now initiated on four infliximab infusions coupled with 18 HBOT sessions and anti-MAP therapy (Table 1). After 6 weeks of this combination therapy the patient reported resolution of clinical symptoms and was passing one to two formed motions per day. Fistula healing was confirmed by inspection and via colonoscopy, with what appeared to be a healing remnant fissure (Figure 6).

### Case 5

A 40-year-old female presented with CD symptoms of rectal bleeding, urgency, fatigue, anemia and three perianal fistulae. Her fistulae were previously treated with long-term ‘setons’ accompanied by various treatments including infliximab without complete healing. The patient was initiated on anti-MAP therapy before the addition of 21 HBOT sessions and three doses of infliximab. She experienced dramatic improvement permitting removal of ‘setons’ and complete healing of all fistulae. Progressive CD remission occurred within 4–6 weeks. A marked reduction in stool frequency was also observed to one formed stool per day from her baseline of >10 stools per day. There was no associated urgency, pain or tenesmus. She has remained asymptomatic without recurrence of fistulae over the last 7 years on a maintenance dose of anti-MAP therapy alone.

### Case 6

A 38-year-old female presented with markedly active CD (CDAI of 416) and an unresolving deep rectolabial fistula that had been present for over a year, despite mesalazine, prednisolone and azathioprine therapy, later infliximab. Colonoscopy revealed extensive ulceration, contact bleeding, pus and inflammation in the left colon. Combination therapy included 21 sessions of HBOT, which led to complete fistula healing, which was confirmed via MRI. The patient also experienced improvement in CD symptoms, reporting two to three soft, formed stools daily, with an absence of blood, fecal urgency and tenesmus. Her CDAI score reduced to 15.

### Case 7

A 27-year-old male presented with four fistula tracts requiring setons *in situ* due to numerous intractable fistulae and abscesses requiring extensive (nine) surgical intervention intravenous antibiotics, anti-inflammatory drugs and infliximab (Figures 7 & 8). The patient had an initial CDAI of 501, with weight loss of 29 kg, and severe anal and pelvic pain. He was commenced on anti-MAP therapy combined with infliximab, and experienced symptomatic improvement. The fistula tracts healed slowly until his HBOT appointment became available for 20 sessions, during which his fistulae progressively healed. At 1-year review, his CDAI score was 76 and repeat examination and colonoscopy revealed completely healed mucosa and fistulae (Figures 9 & 10).

### Case 8

A 40-year-old male with a 16-year history of CD presented with active CD with a CDAI score of 365, and a persistent anorectal fistula. An MRI showed two small perianal fistula tracts. After three doses of infliximab, 21 courses of HBOT and anti-MAP oral antibiotics he improved rapidly and at 6-month follow-up, the fistulae were healed accompanied by complete macroscopic healing of the bowel at colonoscopy.

### Case 9

A 52-year-old male presented with complex perianal Crohn's Disease, which involved a fistula in the intersphincteric space with a small collection in the lower-third of the rectal wall. The patient experienced chronic perianal sepsis as a result, which was resistant to treatment and drainage (Figures 11 & 12). A partial proctocolectomy was performed. Following one years’ treatment with anti-MAP therapy, eight courses of infliximab and 21 sessions of HBOT. The patient underwent an ileorectal reconnection, with complete healing of CD and resolution of fistula, and remains on anti-MAP maintenance therapy alone (Figures 13 & 14).

## Discussion

In this difficult-to-treat population of patients with severe, active CD, who had previously failed conventional therapies (including 5ASA, steroids, infliximab, thiopurines and anti-MAP), combination therapy with infliximab, HBOT and anti-MAP therapy achieved 100% fistulae healing rates in this small cohort. This healing persisted for an average of 18 months with continuation of anti-MAP but on stopping anti-MAP fistulae returned in one patient. This appears to be higher than comparable data for each of the three therapies individually, and indeed any available treatments [10,16–17]. Fistulizing CD frequently presents a therapeutic challenge and significantly impacts on patients’ quality of life. Certain medications that are useful for the treatment of active Crohn's disease may be counterintuitive in the treatment of CD fistulae (e.g., corticosteroids), and can in fact be detrimental [18]. Biologics such as infliximab have improved fistulae treatment – otherwise most cases are intractable and require ‘seton’ placement to prevent abscess formation. While the initial response to infliximab can be dramatic, the median duration of fistula closure is approximately 3 months [19]. In the initial infliximab Phase II and subsequent Phase III trials, 56–68% of fistulae initially healed in 296 patients [1,2]. In the ACCENT 2 trial, there was a 60% response rate of fistulae after 14 weeks. However, on an intention-to-treat basis, at one year only approximately 20% of fistulae healed in patients treated with infliximab [8]. This leaves the vast majority of patients experiencing continued fistulizing activity, with a subgroup of these patients progressing to proctectomy and a permanent stoma [16,18]. In addition, not all fistulae respond to infliximab. Rectovaginal fistulae have a poor response to infliximab alone, achieving initial healing rates of 14–30% compared with 46–78% for perianal fistulae [19].

In our small study, using the combination of infliximab, HBOT and anti-MAP therapy achieved high healing rates of perianal fistulae, including rectovaginal fistulae. Patients had severe CD and were refractory to a number of therapies, including infliximab and anti-MAP therapy alone. Not only did they experience resolution of fistulae, but all nine patients experienced an improvement in CD symptoms. These patients had failed infliximab with or without thiopurines in the past. Given infliximab also has anti-MAP activity [20] we decided to use a short course of infliximab (three to eight infusions) to accelerate healing and continue with HBOT and the antibiotics, as we felt longer term immune suppression may be counterproductive to antibiotic effectiveness. The synergistic effect experienced in these patients may be explained by the differing and potentially complementary actions of these therapies. Treatment of CD using anti-MAP antibiotics with known intracellular activity against MAP, such as rifabutin and clarithromycin, have been shown to induce prolonged remission of CD, and at times deep, longitudinal scarring associated with more profound healing [21]. Similarly, Borody *et al*. have shown fistula healing in three out of five patients treated with anti-MAP therapy [10]. Adverse effects of anti-MAP therapy include redness of body fluids, red tan effect of the skin, arthralgia, transient leucopenia and elevated liver function tests, as the most common and rarely uveitis. An encouraging aspect is the relative low incidence of side effects from these drugs. Typically red discoloration of secretions and skin occur in all patients, while transaminase elevation, leucopenia, uveitis and fevers are largely avoided by a step-up dosing approach.

Due to their location, perianal fistulae are frequently prone to coexisting infection as a result of stool bacterial contamination. Fistulae are proposed to persist due to ongoing ischemia and persistent colonization with anaerobic microorganisms [22]. HBOT is a novel treatment targeting anaerobic bacteria colonizing CD fistulae. Due to the mechanism of action of HBOT, its potential value in fistulae treatment has previously been recognized [23]. Higher atmospheric oxygen environment during HBOT creates unsuitable conditions for anaerobe survival. HBOT also inhibits active inflammation, in part by suppressing TNF-α, IL-1 and IL-6 and enhancing host antibacterial responses [16,24]. Furthermore, HBOT decreases neopterin, myeloperoxidase activity and oxidative stress markers, whilst stimulating angiogenesis [25]. Sporadic case reports of Crohn's perianal disease achieving complete healing on HBOT and non-anti-MAP antibiotics exist [26]. Two uncontrolled prospective studies have been conducted involving a subset of refractory CD patients. In one, ten patients with refractory perianal Crohn's disease underwent 90 min daily sessions for 20 days at a pressure of 2.5 ATM. After two courses, 50% of patients experienced complete healing, with a three [rd] course increasing this rate to 70%. At 18 months follow-up there was no evidence of recurrence [27]. Similar results were shown in another small trial [28]. Brady *et al*. reported a patient with an 8-year refractory CD fistula that healed with one session of HBOT [22]. Minimal adverse effects also exist with HBOT [29].

It is therefore likely that a combination of these diverse mechanisms enabled the profound healing of fistulae seen in these patients, and allowed the subsequent effective maintenance of CD with anti-MAP therapy alone. The effect of the combination therapy generally became evident soon after commencement of the therapy, and such early response could be used to indicate surgery can be avoided. One of the major limitations of this study lies in the use of all three treatments (IFX, HBOT and anti-MAP) concurrently. Hence, it is difficult or even impossible to assess which of these treatments is or are particularly responsible for results of such quality. It is possible that only two of the three treatments used together would provide similar results. Also, this is not a prospective study but all-comer consecutive patients are reported, variations existed among patients, for example, severity of the Crohn's disease, duration of each treatment, number of HBOT sessions and antibiotic regimens (Table 1). This heterogeneity and small patient numbers limit the conclusions of this study. The primary goal of fistula healing was achieved in all patients. Differences in pretreatment severity of the disease to a large extent accounted for the differing treatment durations.

## Conclusion

Our results demonstrate that combining infliximab with anti-MAP therapy and HBOT can achieve a high healing rate of perianal fistulae in Crohn's disease, including rectovaginal fistulae. The use of such combination treatment is a viable option for the treatment of persistent and recurrent fistulae. Future studies using larger sample sizes will enable analysis of fistula characteristics which could predict long-term healing with this combination of therapies.

**Table 1.** **Baseline patient characteristics.**

**Age (years)**	**Fistulae**	**Prior treatment**	**Infliximab 5 mg/kg doses (n)**	**Anti-MAP**	**HBOT sessions**	**Fistulae response**
24	Multiple rectum-to-rectum fistulae and abscesses	Azathioprine, EUAs and seton drainage Metronidazole Infliximab Mesalazine	3	Rifabutin Clofazimine Clarithromycin Ethambutol Ciprofloxacin Metronidazole	30	Complete healing. Healed mucosa persisted at 20-month review
32	High left-sided posterior and right superficial anterior perianal/rectal fistula	Seton insertion/EUA Azathiopurine and infliximab	3	Rifabutin Clofazimine Clarithromycin Ethambutol Ciprofloxacin Metronidazole	30	Complete healing. Healed mucosa persisted at 10-month review
32	Rectovaginal	Infliximab and metronidazole	4	Rifabutin Clofazimine Clarithromycin Ethambutol Metronidazole	21	Complete healing + healed mucosa. Absence of rectovaginal gas passage
31	Right intersphincteric fistula and a posterior anal fissure	Azathioprine infliximab previous bowel resection	4	Rifabutin Clofazimine Clarithromycin Ethambutol Metronidazole	18	Complete healing with healed mucosa. Posterior fissure remained, minimal in size at 6 weeks
20	Three perianal fistulae	Mesalazine prednisone infliximab anti-MAP tx alone	3	Rifabutin Clarithromycin Clofazimine Ethambutol Ciprofloxacin Azathioprine	21	Complete healing. Healed mucosa, remained healed at 8-year follow-up, without setons
38	Deep rectal fistula located below labia	Mesalazine Azathioprine Prednisolone Infliximab seton *in situ*	4	Rifabutin Clofazimine Clarithromycin Ethambutol Metronidazole	20	Complete healing. Rectal fistula had resolved. No seton. Small healed introital tract
27	Four open fistula tracts	Multiple setons *in situ* Failed EUA and fistulotomy Infliximab steroids	6	Rifabutin Clofazimine Clarithromycin Ethambutol Metronidazole	20	Partial closure of fistulae tract on MRI. No discharge or pain. Perianal abscess persisted then resolved
40	Two perianal fistulae	EUA and seton drainage and metronidazole Infliximab	5	Rifabutin Clofazimine Clarithromycin Ethambutol Metronidazole	20	Healed fistulae
52	Complex intersphincteric fistulae. Lower rectal wall abscess	Multiple EUA setons Metronidazole Infliximab	8	Rifabutin Clofazimine Clarithromycin Ethambutol Metronidazole	21	Complete healing of fistulae

Final doses of ramped-up anti-MAP: rifabutin 300 mg twice daily, clarithromycin 1 gm twice daily, clofazimine 100 mg once daily, ethambutol 800 mg once daily. Extra antibiotics for 3–6 months initially: metronidazole 400 mg twice daily, ciprofloxacin 500 mg twice daily.

EUA: Examination under anesthesia; HBOT: Hyperbaric oxygen therapy; MAP: *Mycobacterium avium ss paratuberculosis*; tx: Treatment.

**Executive summary**

Crohn's disease can be complicated by fistulae development between the rectum and the external skin of the anus, and from the rectum to the vagina in more than 20–40% of patients. This is a distressing and chronic complication with poor response to current therapies.
The arrival of the immunomodulator, infliximab (Remicade) has improved healing with initial rates being 60% but in the long-term healing falls to below 40% in spite of administering Remicade.
Oxygen therapy in a hyperbaric chamber (hyperbaric oxygen therapy) and special combinations of antibiotics targeting *Mycobacterium avium ss paratuberculosis* (MAP) have also been shown to heal fistulae but in no more than 30–50% of patients. Combining these partially effective treatments has not been reported.
This report describes the use in nine consecutive patients of a combination of infliximab, hyperbaric oxygen and anti-MAP therapy used together, to see if greater healing of fistulae is achieved and if maintenance of healing is possible with the anti-MAP component.
Although this study is quite small and future results may see some failures, all first nine patients treated with this method healed their fistulae completely without any discharge and without the need to wearing pads to absorb leakage. This appears to be a combination that is worth working with, perhaps optimizing the use of infliximab and hyperbaric oxygen therapy to further optimize healing rates and improve maintenance of healing with long-term antibiotic usage.
